# Analysis on the stability and evolutionary trend of the symbiosis system in the supply chain of fresh agricultural products

**DOI:** 10.1371/journal.pone.0236334

**Published:** 2020-07-30

**Authors:** Baofeng Sun, Mingyang Ma, Yan Li, Lili Zheng

**Affiliations:** Transportation College of Jilin University, Changchun, China; Sunway University, MALAYSIA

## Abstract

In order to increase the stability of fresh agricultural product supply chain, farmers and enterprises need to evolve into a symbiotic system of supply chain. At the present stage, symbiotic relations and evolutionary trends in a symbiotic system for fresh agricultural product supply chains lack quantitative methods for determining symbiotic criteria. In the sense of quantification -oriented criteria, symbiotic systems for fresh agricultural product supply chains are defined, and an improved stationary state analysis method is proposed. Three key steps in this method are quantifying a symbiotic energy model with an evaluation model of ecological carrying capacity, setting up a system evolution model based on the logistic growth function, and verifying the symbiotic system's singularity and phase transition boundary by Lyapunov indirect method. MATLAB numerical simulation shows that types of singularity and the phase transition boundary of symbiotic system are divided effectively. And in both conditions, infinite exponential growth and convergence to steady state, the mutualism mode is the optimal choice for the symbiotic system we defined, symbiotic relations between farmers and cooperative companies are stable and long-term at this time. Those conclusions provide a reference approach to enhance the overall prospective benefits to the fresh agricultural products supply chain.

## Introduction

Fresh agricultural products refer to primary agricultural and livestock products produced or planted by farmers, without or with a small amount of processing, which should not be kept for too long at room temperature [1]. As the source of the supply chain of fresh agricultural products, farmers have the characteristics of little capital, small-scale production, scattered regions, poor storage capacity and low market dominant position. Processing and marketing enterprises (hereafter referred to as enterprises) are the core functional nodes of the supply chain, which are characterized by large capital, large production scale, centralized factory locations, strong storage capacity and market leadership. They depend on each other, and the cooperation modes such as mutualism and parasitism are diversified. In the circulation of fresh agricultural products, the joint efforts of farmers and enterprises are required to maintain fresh quality or higher value.

Due to the perishable nature of fresh agricultural products, there is a shortage of supply chain value-added space, mutually beneficial and win-win relations are difficult to maintain, and the stability of the supply chain system is poor, which restricts the transformation and upgrading of the supply chain system of fresh agricultural products and the industry. The current research fails to provide the internal motivation and the necessity of symbiosis of the relationship between enterprises and farmers in addition to economic benefits, which makes it difficult to form a real symbiosis relationship due to the lack of scientific quantitative standards for the stable factors affecting the cooperative relationship between enterprises and farmers. Therefore, farmers and enterprises should bear the risk of fresh losses of agricultural products, and the high costs of production and marketing efforts. Encouraging farmers and enterprises to develop a win-win cooperative relationship of benefit sharing and cost sharing, quantifying and analysing the symbiosis of the fresh agricultural product supply chain and its evolutionary laws, and scientifically determining and selecting a suitable symbiosis model have become some of the important issues in realizing the long-term and stable development of the fresh agricultural product supply chain.

Based on the quantization of symbiosis criteria, this paper reviews the quantization model of the symbiosis system, as shown in Section 1. Based on the theory of symbiosis and the logic of the criteria, a new method and steps for the stability analysis of symbiotic systems are proposed in Section 2. The symbiotic system corresponding to the supply chain of fresh agricultural products is defined based on the characteristics of fresh agricultural products in Section 3. The symbiotic energy model generated by the interaction of symbiotic units is constructed and quantified by the ecological carrying capacity evaluation method, as shown in Section 4. The Logistic model is selected to reflect the evolutionary direction of the symbiotic system, and Lyapunov's second method is used to study the evolutionary characteristics of the symbiotic system under different symbiotic modes, to provide quantitative analysis support for promoting the evolution of the symbiotic unit relationship to a mutualism mode, as shown in Section 5. Finally, Section 6 concludes the paper and proposes future research directions.

### Reviews for quantitative models of supply chain symbiosis system

Symbiosis refers to the phenomenon that different species in nature interact and live closely together. The fresh agricultural product supply chain based on the symbiosis theory criteria research on symbiosis and the evolutionary direction, are more focused on a series of symbiotic criteria selection and reasoning, dominated by qualitative research, and there is a lack of support criteria for a quantitative method, as well as a lack of physical models and parameter analysis of the problem [2]; this paper is focused on the quantitative criterion for symbiosis theory, and pays attention to systematic reasoning based on criterion logic, focusing on the key links and improvement points of the symbiotic system stability analysis method and expanding upon the following research review of the same type.

### Research on the method system of the supply chain symbiotic system

(1) SCOR reference model

Ntabe E N et al pointed out that the SCOR reference model mainly focused on the following two parts: firstly, through the establishment of the supply chain nodes interaction process view model, to analyze the interaction between enterprises within the system; secondly, an interface view of the supply chain including enterprises, customers and suppliers is established to reflect the interaction between adjacent nodes [3].

The disadvantage of SCOR reference model is that it is the modeling of the interaction between some nodal enterprises in the supply chain, which is not integrated. From the perspective of content, it is more about the description of the overall operation process of the supply chain, lacking the attention to the characteristics of nodal enterprises.

(2) “X” model

Maerhua et al. proposed the "X" model of enterprise expansion. The "X" model describes a series of logistics-related activities such as production control, cost accounting, delivery planning, assembly control, and capacity demand planning for nodal enterprises in supply chain. The "X" model combines supply, production and sales to reflect the overall benefits [4].

A. W. Scheer added the element of informatization based on the "X" model and innovatively proposed the "X" model with information integration function [5]. Although the practical scope of the "X" model was expanded through functional improvement, the "X” model is lack of quantitative standards and mechanisms in actual operation. It is still difficult to achieve computer modeling and simulation.

(3) Game theory

Using game theory as the theoretical basis to analyze the symbiotic relationship of supply chain node enterprises, the existing literature mainly studies the benefits and costs of the supply chain, the assumption of rational actors and the integrity level of the symbiotic system.

Sun Ying et al. attributed the benefits and costs of the supply chain to the imbalance between transactions and division of labor [6]. In the supply chain, the node companies all aim to maximize their respective interests. On the premise of not weighing the degree of specialization, a reasonable division of labor mechanism has not been established. In order to increase the number of transactions and obtain profits, the irrational game mode is selected, make the whole supply chain profit levels reduced at the same time, increasing the transaction cost, and reduce the overall competitiveness.

Xiaodong Liu and Guixu Zhang pointed out that generally in the game relationship, both parties to the game subject are required to be rational actors, that is, both parties to the game subject aim to maximize their own interests [7]. However, in the symbiosis of the supply chain, more emphasis is placed on maximizing overall benefits and the most stable state of cooperation. Therefore, when combining game theory and symbiosis theory, there is a certain contradiction in the definition of the main agent.

Tongshan Li et al considering the deviation of the above completely rational actors from the goal of the symbiotic unit, proposed a game equilibrium problem based on the integrity level of game theory to solve the symbiotic system. Among them, the premise of the subject's game assumes that both parties are limited rational actors, that is, the goals and expected returns of both parties are determined by the integrity level of the enterprise [8]. Although this hypothesis can solve the combination of game theory and supply chain symbiosis system, there is no scientific quantification standard in the method of judging the corporate integrity level, which makes the results subjective.

In summary, the symbiosis theory has been widely applied to various fields and has contributed to the cooperative relationship and sustainable development of the research objects. In the study of existing supply chain symbiosis systems, qualitative methods are often used to reflect the strength of interaction, and there is a lack of a more scientific quantitative mechanism. The thesis attempts to use fresh agricultural products as the main body of research, and the symbiotic relationship between enterprises and farmers as the research object. By analyzing the essence of the interaction between the two, a symbiotic system of fresh agricultural products management enterprises and farmers is constructed, and the intensity of the symbiotic interaction is quantified, More scientifically and intuitively reflect the essence of the interaction between the two symbiotic units.

#### The symbiotic energy model integrating ecological carrying capacity

Odum defines "generalized ecological carrying capacity " as the maximum number of human activities that can be accommodated by the natural, economic and social complex ecosystem under the premise of coordinated and sustainable development of natural resources, ecological environment, and economic and social sub-items within a certain region [9–11]. Yafen He and Hualin Xie use the ecological footprint method to evaluate the ecological carrying capacity (ECC) of Nanchang city at a city, county and grid scale, respectively [12]. Jiang Peiwen and Rees W et al. used the ecological footprint method to calculate the ecological footprint and ecological carrying capacity of agriculture-related land use in Shaanxi Province in 2015 [13,14]. Cao Zhi believed that the measurement of the ecological footprint is the consumption footprint of agricultural production, that is, the materials needed for agricultural production [15]. This paper refers to the concept of generalized ecological carrying capacity, defines the ecological footprint as the consumption footprint of agricultural resources, and introduces the ecological footprint measurement model into the non-symbiotic energy part of the symbiotic energy model to improve the symbiotic energy model.

#### Logistic symbiotic environmental model considering the change of environmental tolerance

The logistic model, also known as the retarded growth model, is considered to be an optimal mathematical model to describe the population growth rule under limited resource conditions, with rich connotations in ecology, anthropology, zoology and economics [16]; Yingying Tao and Lihong Han described the development law of the green building industrial chain symbiotic system under different symbiosis relationships through a logistic model [17]; Ding Y, Zhou B and Ling D proposed an enterprise symbiosis mechanism and symbiosis stability monitoring model of supply chain alliances based on logistic and Lotka-Volterra equations [18]. In population ecology, environmental capacity is also known as maximum environmental capacity, which is consistent with the concept of the logistic model. In essence, this refers to limited growth in a limited environment. With the help of variable environmental capacity, this paper not only reflects the constraints of environment on species but also reflects the dynamic variables of its own changes. Based on the Logistic growth function, it establishes the symbiosis environmental model that conforms to the relationship between the fresh agricultural product supply chain and environmental capacity, as a criterion of the stability analysis of the quantitative basis. Thus, the variable environmental tolerance characterizes the uncertain fresh quality or higher value of agricultural products that farmers and enterprises are concerned with.

#### Lyapunov indirect method and its application

The Lyapunov stability indirect method determines the stability of nonlinear systems by studying the distribution of characteristic roots of linearized equations of state [19]. Chunmei Liu proposed that the Lyapunov method can be used for the stability analysis of systems without delay terms [20]. Taking nonlinear time-invariant systems as an example, a linearization method for nonlinear systems was expounded. HAMZA, Alaa and ORABY, Karima obtained new sufficient conditions for the multiple stability of nonlinear dynamic equations by using the Lyapunov indirect method [21]. In this paper, the Lyapunov indirect method is applied to the analysis of the stability of symbiotic systems. Through an approximate transformation, the environmental model is transformed into a systematic evolutionary model, and the evolutionary singularity and phase transition boundary of symbiotic systems are judged by the positive and negative characteristics of characteristic roots.

This paper proposes an improved symbiosis system stability analysis method for criteria quantification. This method takes the symbiosis system of the supply chain of fresh agricultural products as the research object and uses systematic reasoning based on criteria logic for the characteristics of fresh agricultural products, i.e., the uncertain quality and value of fresh agricultural products in supply chain circulation.

To summarize, three improvement points we contribute for criterion quantification are shown as follows: First, based on the Logistic growth function, the symbiotic energy model that conforms to the interaction relationship of the fresh agricultural products supply chain and the change of environmental tolerance is reconstructed. Second, the ecological footprint measurement method of the generalized ecological carrying capacity is used to quantify the non-symbiotic energy model. Considering the efforts and profit distribution of fresh agricultural product enterprises and farmers, the new symbiosis energy is measured by the change of expected income before and after the formation of the symbiosis relationship. Last not the least, according to the self-similar approximation theory, the Lyapunov characteristic exponential function is constructed through an approximate transformation to quantify the dynamic system of the symbiotic environment.

The eight basic criteria from the symbiosis theory involved can reflect the interaction of the corresponding symbiosis units in a certain symbiosis mode.

Criterion 1: For the quality parameter compatibility criterion, there must be some factors that can express each other or influence each other between symbiotic units, so that interactions can be formed to affect the symbiotic system;

Criterion 2: The symbiotic tissue model criterion is mainly determined by the frequency of interaction of the symbiotic units;

Criterion 3: The symbiotic behavioural model criterion is mainly determined by the interaction mode and intensity of the symbiotic unit. In different symbiotic behavioural modes, symbiotic energy of different intensities will be generated between the two symbiotic units;

Criterion 4: For the symbiosis energy generation criterion, symbiotic energy is formed by the interaction of symbiotic units, which is the basic condition for the existence and development of symbiotic systems;

Criterion 5: The symbiotic interface selectivity criterion is the choice of the environment in which the symbiotic interaction occurs;

Criterion 6: For the symbiotic phase change criterion, the symbiotic interaction phase change determines the system evolution singularity and boundary value;

Criterion 7: For the symbiotic evolutionary criterion, the system changes phase at a singular point and evolves to a critical state at the boundary;

Criterion 8: For the symbiosis stability criterion, when the symbiotic unit develops at zero speed, the system is in a stable state.

## Materials and methods

### Definition of the symbiosis system in the supply chain of fresh agricultural products

According to criterion 1, the relationship between nodes in the supply chain of fresh agricultural products is reduced to a symbiotic relationship. This is because of the close interdependence between farmers and fresh agricultural product enterprises. In the process of creating a professional division of labour with complementary advantages, integrating the production sources of fresh agricultural products, limiting the loss of fresh agricultural products in transport, and ensuring the quality and safety of fresh agricultural products, a symbiotic relationship has been formed in order to evolve in a more competitive direction.

Furthermore, the symbiotic system of the fresh agricultural products supply chain is established with the core of the symbiotic system consisting of "three elements" (symbiotic unit, symbiotic mode and symbiotic environment) and the combination of criteria 2, 3 and 5. The symbiosis system of the supply chain of fresh agricultural products is an organic whole that enables the symbiotic units to interact with each other in a certain symbiotic environment with a certain symbiotic mode in the process of production, processing, transportation and sales of fresh agricultural products, and enables the symbiosis process to proceed smoothly and develop.

#### Symbiotic unit

In the symbiotic relationship between agricultural product enterprises and farmers, the quality parameters are defined as the interaction between two symbiotic units in the supply and demand of fresh agricultural products, the distribution of profits, and the asset and technology combination in production and processing. From the three aspects of the characteristics of fresh agricultural products, the willingness and efforts of farmers and enterprises, the following three kinds of mass parameter functions are designed to reflect the internal dynamic mechanism of the symbiosis unit.

⑴The fresh loss rate function

Fresh agricultural products have perishable and fragile characteristics. It is assumed that the time elapsed from production to sale to the market during the life cycle *T* is *t*(0≤*t*≤*T*) and that the degree of loss will increase with the increase of time *t*. To describe the attenuation law of fresh agricultural products in terms of loss, the loss rate function of fresh agricultural products is introduced [22]:

$$\lambda (t)=e^{\frac{t\ln2}{T}}-1$$ (1)

⑵The benefit distribution function

To obtain a stable source of agricultural production, the fresh agricultural product management enterprise signs a rebate contract with the farmers to enhance their willingness to work together. The market demand for fresh agricultural products is *x*: due to the loss of fresh agricultural products in transport, the actual sales volume of fresh agricultural products is *q* = (1−*λ*(*t*))*Q*, in which the management enterprises should sign the order with the farmers as *Q*(*Q*≥*x*), the purchase price is *p*_1_, the sales price is *p*_2_. According to the profit situation, the fresh agricultural products management enterprises will repay the profits to the farmers in proportion as (1−*φ*) to the sales. The expected income of the fresh agricultural product management enterprises and farmers under symbiotic conditions are:

The expected income of farmers is:

$$\pi_{1}^{s}=(p_{1}-c_{1}^{s})Q+(1-\varphi )P_{2}Q^{*}$$ (2)

$$Q^{*}=[q-\int_{0}^{q}(q-x)f(x)dx]$$ (3)

The expected income of the enterprise is:

$$\pi_{2}^{s}=\varphi p_{2}[q-\int_{0}^{q}(q-x)f(x)dx]-(p_{1}Q+c_{2}^{s}q)$$ (4)

$c_{1}^{s}$ and $c_{2}^{s}$ are the production cost of farmers and the sales cost of enterprises in the symbiotic mode, respectively. Q* is the actual sales volume of fresh agricultural products. *x* refers to the customer demand. *f*(*x*) refers to the probability density function of random variable *x*.

⑶The effort cost function

The qualified output of fresh agricultural products can be increased by means of input into production efforts, according to Xie Gang and Ji-ca Li [23,24]. Assume that when the production effort of fresh agricultural product farmers is *x*_*f*_(*x*_*f*_>0), the sales volume of qualified fresh agricultural products is *Q*(1+*x*_*f*_), and the production effort cost is $1/2\beta_{f}x_{f}^{2}$. When the sales effort is *x*_*r*_(*x*_*r*_>0), the market price is *p* = *p*_2_(1+*x*_*r*_), and the sales effort cost is $1/2\beta_{r}x_{r}^{2}$.

In the joint efforts of the fresh agricultural product management enterprises and farmers, the enterprise and the farmers are allocated the production effort cost. Assume that the proportion of the enterprises in the production effort cost is *z*, and the price of the purchased fresh agricultural products is *p*_1_. Under the symbiotic state, the expected income of farmers who produce fresh agricultural products under the production effort cost is:

$$\pi_{f}^{s}=\left(p_{1}-\frac{\left(1-z\right)}{2}\beta_{f}x_{f}^{2}-c_{1}^{0}\right)(1+x_{f})Q$$ (5)

The expected return of the enterprises under the cost of sales and the cost of effort allocated is:

$$\pi_{r}^{s}=p_{2}(1+x_{r})[q-\int_{0}^{q}\left(q-x\right)f(x)dx]-\left[(p_{1}+\frac{z}{2}\beta_{f}x_{f}^{2})Q+(c_{2}^{0}+\frac{1}{2}\beta_{r}x_{r}^{2})q\right]$$ (6)

Among them:

$$\left\{ \begin{array}{c} c_{1}^{s}=c_{1}^{0}+\frac{\left(1-z\right)}{2}\beta_{f}x_{f}^{2}\\ c_{2}^{s}=c_{2}^{o}+\frac{z}{2}\beta_{f}x_{f}^{2}+\frac{1}{2}\beta_{r}x_{r}^{2} \end{array}\right.$$ (7)

$c_{1}^{s}$ and $c_{2}^{s}$ are the total cost functions of the fresh agricultural product management enterprises and the farmers in the symbiotic state, considering the effort costs of production and sales.

#### Symbiotic mode

The symbiotic interface between farmers and enterprises is the market of fresh agricultural products. On this interface, farmers are interdependent and mutually beneficial, exchanging materials, information and energy to form new symbiotic energy. It is consistent with the basic feature of natural symbiosis that "new energy is generated by mutual influence, without affecting its own structure and nature". Due to the complex and changeable market, it is necessary to ensure the continuous operation of the symbiosis system by the close interaction between the two symbiosis units, which is dominated by the core enterprise and relies on the credit mechanism. By referring to the symbiosis model of insect species in nature, which is parasitic, partial symbiosis and mutualistic, the two symbiosis units in the supply chain of fresh agricultural products are dominated by a mutualism and parasitism model.

#### Symbiotic environment

The symbiotic units interact with each other in the system environment until they reach a steady state. Due to an abrupt change of the system environment or other influencing factors, the system will be shaken. When the critical value of a certain condition is reached, the system state will evolve into a different state. In view of the variable environmental capacity, it not only reflects the restriction of the environment on species but also reflects the dynamic variable characteristic of its own change. The symbiotic system based on variable environmental capacity has undergone an evolutionary process of generation, development and stability, and it conforms to the logistic growth function, which can be expressed as:

$$\frac{dN_{i}}{dt}=\gamma_{i}N_{i}-\frac{\gamma_{i}N_{i}^{2}}{K_{i}}$$ (8)

*N*_1_ and *N*_2_ represent the economic output indicators of fresh agricultural products management enterprises and farmers within a symbiotic relationship, respectively; *γ*_*i*_ represents the phylogenetic rate of the two symbiotic units; and *K*_*i*_ is the environmental capacity of the fresh agricultural product management enterprises and farmers in the symbiosis system, which serves as their symbiosis energy *E*_*i*_.

### Quantitative model of the fresh agricultural supply chain symbiosis system

According to criterion 4, the symbiotic energy model of the fresh agricultural products supply chain symbiotic system, namely, Eq (9), was refined, and then the symbiotic system evolution model, namely, Eq (26), was established to support the stability analysis of the symbiotic system.

#### Symbiotic energy model

Symbiotic energy *E*_*i*_ can be defined as follows: without symbiosis, their respective energy is *A*_*i*_ and newly added energy in symbiosis is *S*_*i*_, as shown in Eq (9)

Without symbiosis, system energy *A*_*i*_ is subdivided into *A*_*1*_ and *A*_*2*_ with the help of the concept of functional ecological carrying capacity under natural conditions and the measurement method of agricultural production and consumption footprint. Set *μA* to convert ecological carrying capacity into economic indicators to unify dimensions. *S*_*i*_({*N*_1_,*N*_2_,⋯,*N*_*i*_}) represents the new energy created by fresh agricultural products enterprises and farmers under the symbiotic conditions, *S*_*i*_({*N*_1_,*N*_2_})≥0. *B*_*i*_∈(−∞,+∞) is the intensity of the species' acceptance of new energy.

$$E_{i}=\mu A_{i}+B_{i}S_{i}(\left\{N_{1},N_{2}\right\})$$ (9)

Among them:

$$\mu A_{i}>0$$ (10)

$$S_{i}(\left\{N_{1},N_{2}\right\})\geq 0$$ (11)

$$B_{i}\in (-\infty ,+\infty )$$ (12)

(1) Ecological footprint of fresh agricultural inputs of farmers:

$$A_{1}=\sum_{i=1}^{n}\frac{C_{i}}{Y_{i}}$$ (13)

Where *C*_*i*_ represents inputs of farmers of *i* type of fresh agricultural products, and *Y*_*i*_ represents the regional average output of inputs of *i* type.

(2) Ecological footprint of fresh agricultural products enterprises:

$$A_{2}=\sum_{i=1}^{n}\frac{l*C_{i}}{Y_{i}}$$ (14)

Where *l* is the amount of labour force in the enterprise, *C*_*i*_ is the per capita labour value of *i* fresh agricultural products, and *Y*_*i*_ is the average regional output of *i* fresh agricultural products.

From the perspective of the economic interest relationship between fresh agricultural products enterprises and farmers, the newly increased symbiotic energy *S*_*i*_ is quantified as the change amount of expected earnings before and after the formation of a symbiotic relationship. Let *i* = 1,2 represent farmers and enterprises, respectively, *i* = *o*,*s* represents non-symbiotic and symbiotic conditions, respectively, then the following expression represents the expected benefits of fresh agricultural products management enterprises and farmers under non-symbiotic conditions:

$$\sum_{i=1}^{2}\pi_{i}^{0}=\pi_{1}^{0}+\pi_{2}^{0}$$ (15)

Among them:

$$\pi_{1}^{0}=(p_{1}-c_{1}^{0})Q$$ (16)

$$\pi_{2}^{0}=p_{2}[q-\int_{0}^{q}(q-x)f(x)dx]-(p_{1}Q+c_{2}^{0}q)$$ (17)

q=(1−λ(t))Q (18)

(16) and (17) are the expected returns of farmers and enterprises under non-symbiotic conditions, respectively; (18) *q* is the actual shipment amount of the operating enterprises' procurement; here, the loss ratio function *λ*(*t*) is Eq (1).

Under the symbiotic condition considering the efforts and profit distribution of fresh agricultural products enterprises and farmers, the expected benefits of the two are:

$$\sum_{i=1}^{2}\pi_{i}^{s}=\pi_{1}^{s}+\pi_{2}^{s}$$ (19)

$$\pi_{1}^{s}=\left(p_{1}-\frac{\left(1-z\right)}{2}\beta_{f}x_{f}^{2}-c_{1}^{0}\right)(1+x_{f})Q+(1-\varphi )p_{2}(1+x_{r})Q$$ (20)

$$\pi_{2}^{s}=\varphi p_{2}(1+x_{r})[q-\int_{0}^{q}(q-x)f(x)dx]-\left[(p_{1}+\frac{z}{2}\beta_{f}x_{f}^{2})(1+x_{f})Q+(c_{2}^{0}+\frac{1}{2}\beta_{r}x_{r}^{2})q\right]$$ (21)

$$Q^{*}=[q-\int_{0}^{q}(q-x)f(x)dx]$$ (22)

In summary, combined with the above definition of *S*_*i*_({*N*_1_,*N*_2_}), we can obtain:

$$S_{i}(\left\{N_{1},N_{2}\right\})=\sum_{i=1}^{2}\left(\pi_{i}^{s}-\pi_{i}^{0}\right)$$ (23)

#### Symbiotic system evolution model

The evolution direction of the symbiotic system depends on the change of the symbiotic dynamic system and its energy. It is necessary to construct a set of ordinary differential equations of the dynamic system to describe the symbiotic environment based on Eq (8), to realize the quantification of the symbiotic phase change criterion, symbiotic evolution criterion and symbiotic stability criterion. Assume the following:

(1) *x* and *z* are defined as the economic output of farmers and enterprises, respectively. Then, $\frac{dx}{dt}$ and $\frac{dz}{dt}$ represent the trend indicators of the economic output of farmers and enterprises, respectively.

(2) *y*_*i*_ is defined as the total symbiotic energy of farmers and enterprises, where *a*_*i*_ is set as the ecological carrying capacity of farmers and enterprises under non-symbiosis conditions, and *B*_1_ = *b*. *B*_2_ = *g* is set as symbiosis parameters of farmers and enterprises, respectively. It can be known from the symbiosis mode of fresh agricultural product management enterprises and farmers that when *b*>0,*g*>0, the two symbiosis units are in a mutual-symbiosis mode. When *b*>0,*g*<0 or *b*<0,*g*>0 or *b*<0,*g*<0, the two symbiotic units are in parasitic mode, and other situations are inconsistent with reality, which will not be discussed in this paper.

(3) set *xz* to represent the new energy added by farmers and enterprises under the symbiosis condition, then *bxz* represents the effect of farmers affected by symbiosis, and *gxz* represents the effect of operating enterprises affected by symbiosis.

In conclusion, the symbiotic energy model of fresh agricultural farmers and management enterprises can be expressed as follows:

$$y_{1}=a_{1}+bxz,y_{2}=a_{2}+gxz$$ (24)

The symbiotic environmental model between fresh agricultural products management enterprises and farmers can be expressed as follows:

$$\left\{ \begin{array}{c} \frac{dx}{dt}=x-\frac{x^{2}}{a_{1}+bxz}\\ \frac{dz}{dt}=z-\frac{z^{2}}{a_{2}+gxz} \end{array}\right.$$ (25)

where in order to make the ordinary differential equation solvable, it should be set that *x* = *x*(*t*),*z* = *z*(*t*), in Eq (25) has the initial solution *x*(0) = *x*_0_,*z*(0) = *z*_0_ and the changes of variables in the system are symmetric.

The paper is based on the self-similar approximation theory [25]. When *n*→+∞, there are ${\left(1+\frac{a}{n}x\right)}^{n}\to e^{ax}$, the two equations have the same mathematical characteristics and evolutionary direction. Therefore, the symbiotic system environment model (25) of the fresh agricultural product management enterprises and the farmers is transformed into a symbiotic system evolution model (26), which can be used as the Lyapunov characteristic exponential function to be constructed:

$$\left\{ \begin{array}{c} \frac{dx}{dt}=x-x^{2}e^{-bxz}\\ \frac{dz}{dt}=z-z^{2}e^{-gxz} \end{array}\right.$$ (26)

Among them, the symbiotic parameter in the symbiotic system is *b*,*gϵ*(−∞. +∞); the initial solution of the symbiotic system evolution is *x*(0) = *x*_0_,*z*(0) = *z*_0_; the requirement is *x*(*t*)≥0,*z*(*t*)≥0.

*Singularity distribution and phase change boundary determination of the symbiotic system*. Based on the evolution model of the symbiotic system (26) and criteria 6, 7 and 8, the singularity distribution and phase transition boundary determination method of the symbiotic system based on Lyapunov are presented in this section. According to criterion 8, in order to maintain the stability of the symbiotic system between fresh agricultural products enterprises and farmers, the rate of change of economic output of the system is zero. Set $\frac{dx}{dt}=\frac{dz}{dt}=0$ and set the non-zero stable solution {x*≠0,z*≠0} as the solution of the system evolution model (26):

$$\left\{ \begin{array}{c} x^{*}=e^{bx^{*}z^{*}}\\ z^{*}=e^{gx^{*}z^{*}} \end{array}\right.$$ (27)

From ${\left(x^{*}\right)}^{g}={\left(z^{*}\right)}^{b}=e^{bgx^{*}z^{*}}$, formula (27) can be expressed as:

$$\left\{ \begin{array}{l} x^{*}=exp(b{\left(x^{*}\right)}^{1+\frac{g}{b}})\\ z^{*}=exp(g{\left(z^{*}\right)}^{1+\frac{b}{g}}) \end{array}\right.$$ (28)

A Taylor expansion of the system Eq (26) is used to obtain an approximate Lyapunov system of equations, and the two characteristic root expressions are obtained as follows:

$$\left\{ \begin{array}{c} \lambda_{1}=-1\\ \lambda_{2}=-1+(b+g)x^{*}z^{*} \end{array}\right.$$ (29)

**Fig 1:**
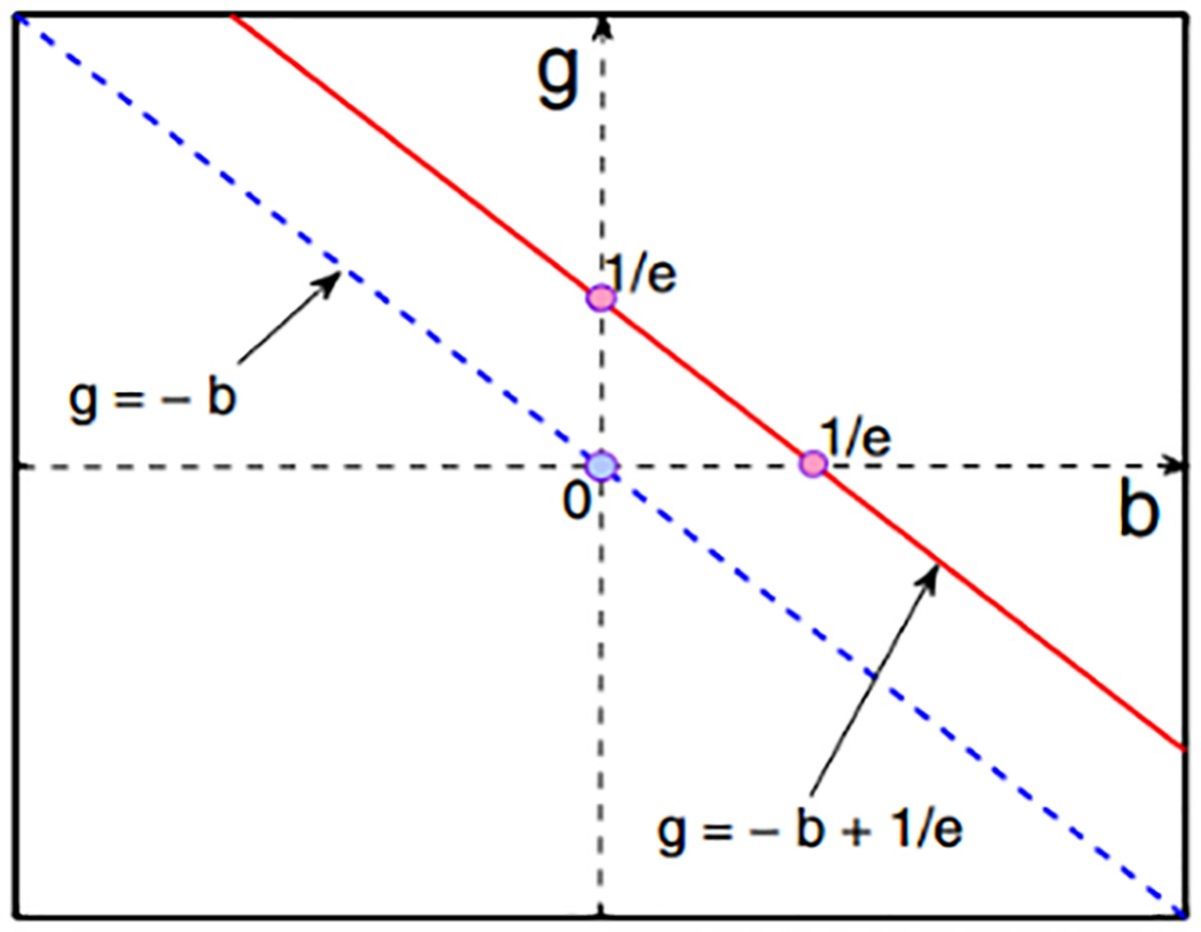
Evolution singularity and boundary of symbiosis system between fresh agricultural product management enterprises and farmers.

As shown in Fig 1, the positive and negative values of *λ*_1_ and *λ*_2_ are used to determine the types of singularities of the symbiotic system between fresh agricultural enterprises and farmers.

The boundary of phase transition is the red line $b+g=\frac{1}{e}$ and the blue line *b*+*g* = 0, which divides the evolution of the symbiotic system into three domains.

(1) There is no stable point on the upper side of the phase transition boundary $b+g=\frac{1}{e}$ along the symbiotic system. At this time, the evolution of the symbiotic system is in an infinite exponential growth trend.

(2) There is a stable point along the lower boundary of the phase transition boundary *b*+*g* = 0 of the symbiotic system, that is, there is a unique solution when the evolution rate of the symbiotic system is zero, indicating that the system converges to steady state at this time.

(3) There are two stable points in the middle of the two-phase transition boundary, namely, stable degenerate points and saddle points. At this time, there are two solutions when the system evolution rate is zero, indicating that the symbiotic system is in an evolutionary state.

## Results and discussion

### Convergence analysis of a steady state of the symbiotic system in the fresh agricultural product supply chain

#### Urban rail transit network and basic data

The evolutionary state of the symbiotic system of Eq (26) is divided into two cases: infinite exponential growth and system convergence to steady state. Based on the 3.3 singular point distribution and phase change boundary determination method, this section compares and analyses the evolutionary direction of the system of different symbiotic parameters and symbiotic modes. It provides quantitative analysis for promoting the evolution of the symbiotic unit to the mutualism symbiotic model.

Numerical Simulation Tool: MathWorks MATLAB 2014b

Simulation running environment: windows 64 bit for windows 7

Simulation step: t = 0.5

Initial conditions: {*x*_0_ = 0.01,*z*_0_ = 5};

Experiment 1: infinite exponential growth: $b+g>\frac{1}{e}$;

Contrast parameters: {*b* = 2,*g* = 1}_1_ and {*b* = −0.8,*g* = 1}_2_;

Experiment 2: convergence to steady state: $0\leq b+g\leq \frac{1}{e};\;b+g<0;$

Symbiotic contrast parameter:{*b* = 0.25,*g* = 0.1}_1_; {*b* = −0.75,*g* = 1}_2_; {*b* = −1,*g* = −2}_3_.

Through experiments 1 and 2, the logarithmic form of solution *x*(*t*)≥0,z(*t*)≥0 of two symbiotic units is ln*x*(*t*),ln*z*(*t*), and the evolutionary direction of ln*x*(*t*),ln*z*(*t*) at time *t*→∞ is compared.

#### Evolutionary trend of the symbiotic system under infinite exponential growth

**Fig 2:**
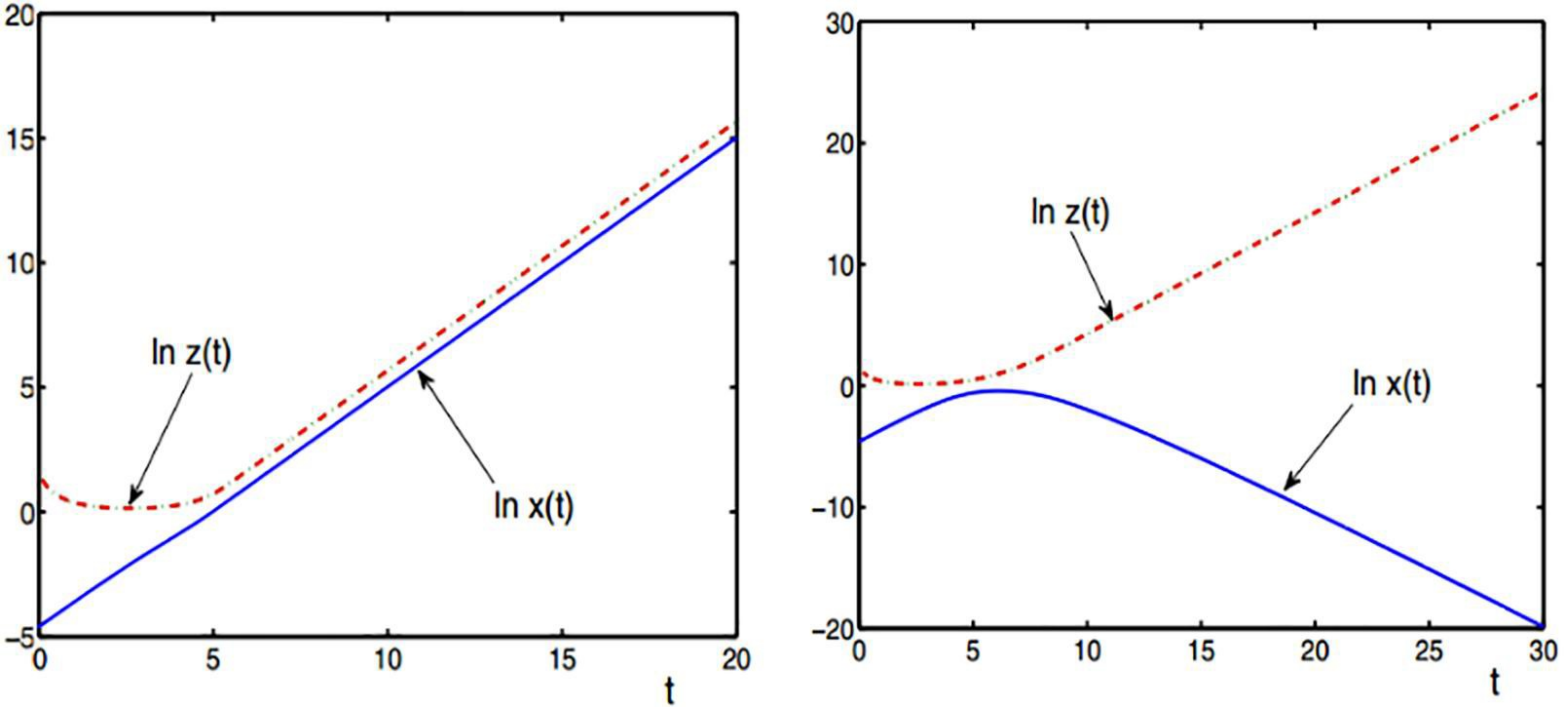
The infinite exponential growth of symbiotic systems. (a) *b*>0,*g*>0, (b) *b*<0,*g*>0.

The simulation results of experiment 1 are shown in Fig 2. Under the same initial conditions, with time *t*→∞, the development speed of (a) the symbiotic unit shows an infinite growth phenomenon and eventually exceeds the initial value. (b) the development speed of (b) symbiotic units shows unlimited growth, while the other side shows a negative growth direction after a certain moment and will eventually be lower than the initial value.

Experimental data show that the symbiosis parameter *b*,*g*>0, which is the mutualism mode state at this time, will effectively drive and maintain the infinite growth state of the whole symbiosis system. In contrast, *b*<0,*g*>0 is a parasitic mode state, which will eventually lead to a decrease in the rate of evolution of the symbiotic system, or even negative growth, leading to the collapse of the symbiotic relationship between the two.

#### Evolutionary trend of symbiotic systems converge to steady state

**Fig 3:**
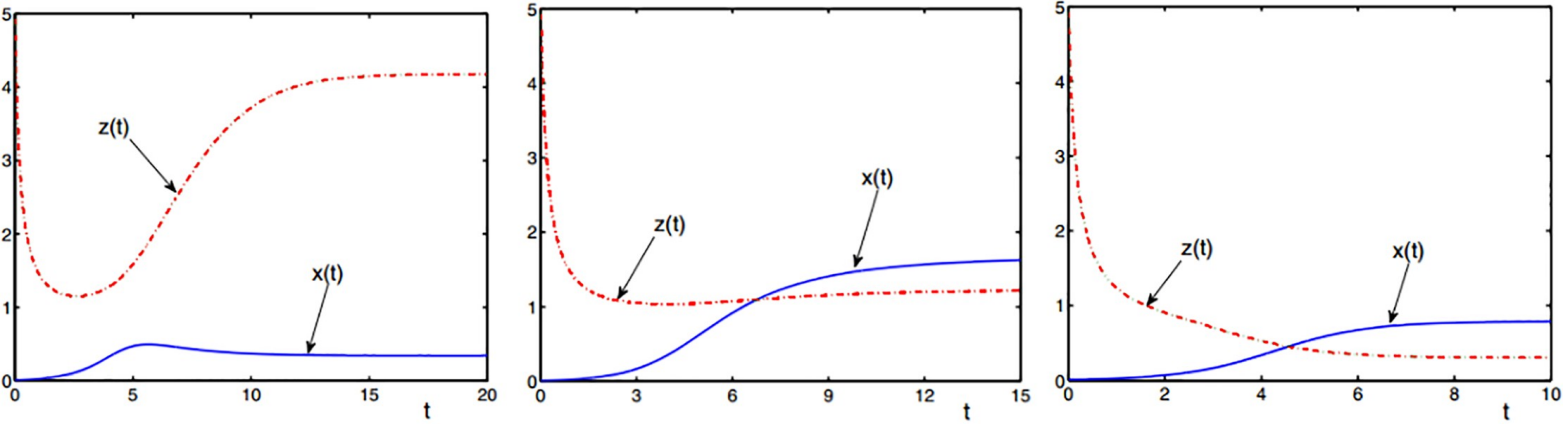
The symbiotic system converges to a steady-state trend. (a) *b*,*g*>0, (b) *b*<0,*g*>0, (c) *b*<0,*g*<0.

The simulation results of experiment 2 are shown in Fig 3. When the symbiotic parameter satisfies $0\leq b+g\leq \frac{1}{e}$, Fig 3A reaches steady state at *t* = 20 and symbiotic parameter *b*,*g*>0; in Fig 3B, steady state is reached at *t* = 15, and symbiotic parameter *b*<0,*g*>0 is obtained. Compared with the system in Fig 3B, the system in Fig 3A has a slower convergence speed, and the difference between the initial state and the stable point is small. The overall benefit of the symbiotic system in Fig 3A is greater than that in Fig 3B. when the symbiotic parameter satisfies *b*+*g*<0 and *b*<0,*g*<0, Fig 3C reaches steady state at *t* = 10. The convergence rate of the system in Fig 3C is significantly higher than that in Fig 3A and 3B, and there is a big difference between the initial state and the stable point, and the overall system income is the least in the three scenarios.

### Case study: Stability and evolution direction analysis of the symbiotic system

This section calculates and verifies the effectiveness of the symbiosis energy model through a symbiosis system calculation example of blueberry enterprise A and planting farmers in Changbai Mountain. Based on the improved stability analysis method of the symbiotic system proposed for criterion quantification, the evolution direction of the symbiotic system under different symbiotic modes is analysed and judged.

#### Effectiveness analysis of the symbiotic energy model of the symbiotic system

**Fig 4:**
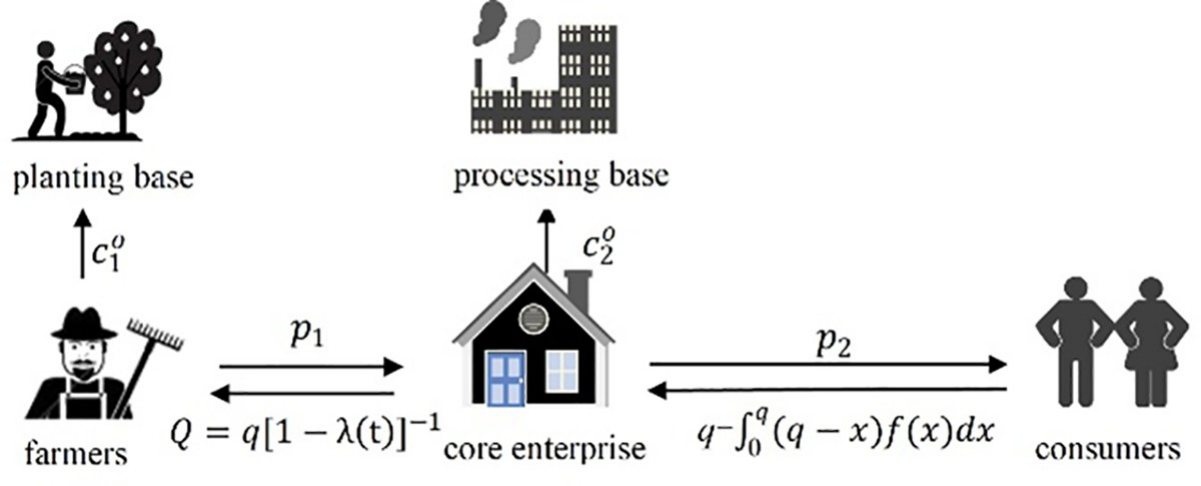
The operation mode of fresh agricultural products enterprises and farmers without symbiosis mode.

The paper takes the A-enterprise of an ecological technology company in Jilin Province as a symbiotic unit that cooperates with farmers. Company A is a fresh fruit enterprise at Changbai Mountain in the field of blueberry processing and product sales. It has 1800 acres of blueberry as its own specialty planting base, 9500 acres of regional chain planting base, and drives more than 1,230 farmers around the country. No symbiosis is involved, and the operation mode of enterprise A and surrounding farmers is shown in Fig 4.

Farmers planted blueberries at Changbai Mountain at the cost of $c_{1}^{0}$ for the planting base, and sold an amount of blueberries *Q* to enterprise A at the price of *p*_1_. After the lean processing of blueberries at the cost of $c_{2}^{0}$, enterprise A sells blueberries to consumers at the price of *p*_2_. Where consumer demand is *x*, then the sales volume of enterprise A is an integral variable related to *x*.

**Fig 5:**
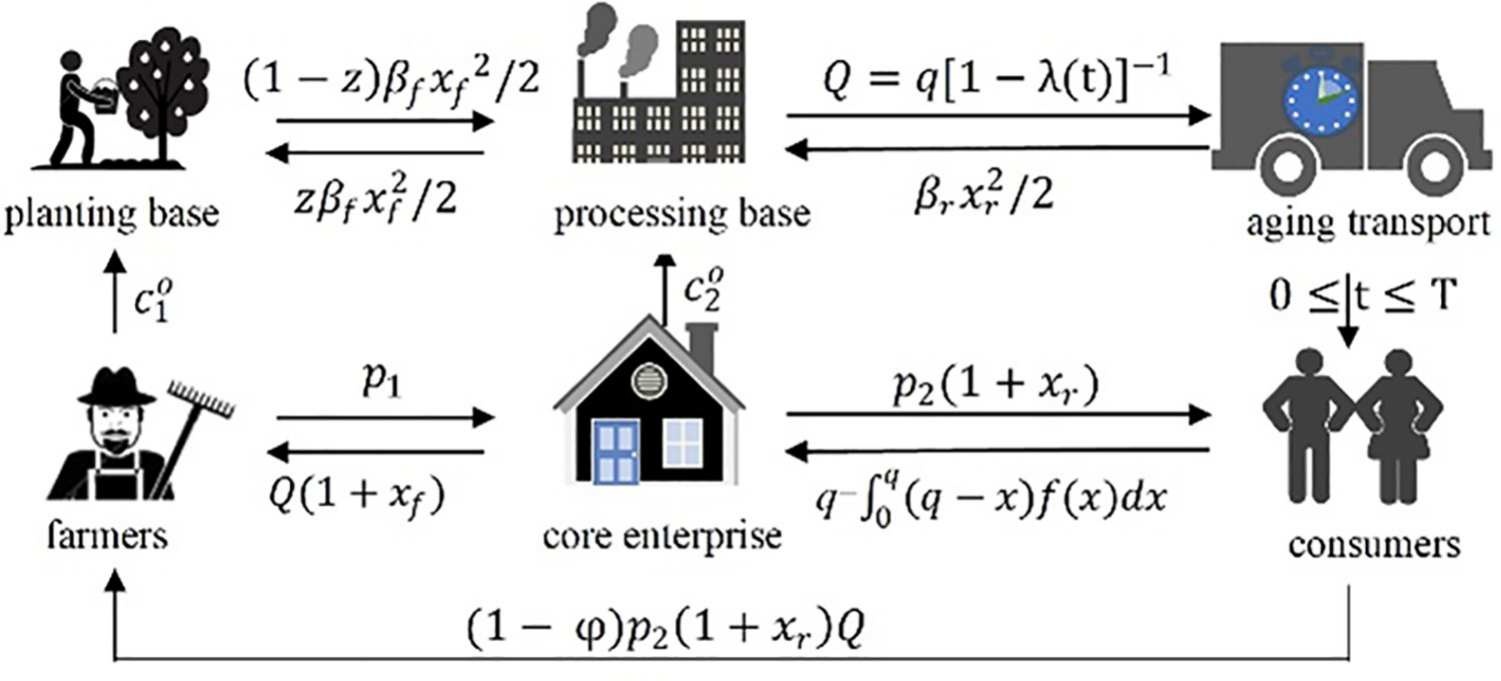
The symbiosis mode of fresh agricultural product management enterprises and farmers under the symbiosis mode.

Under the symbiotic condition, the interaction between enterprise A and the surrounding farmers is shown in Fig 5.

To maintain a good symbiotic relationship, in terms of production and planting, farmers will share the effort cost of *z* to the processing cost of the enterprise, that is, farmers will produce Changbai Mountain blueberries at the cost of $c_{1}^{0}+(1-z)\beta_{f}x_{f}^{2}/2$ in the planting base, and increase the order quantity of blueberries of enterprise A to *Q*(1+*x*_*f*_) through production efforts, and sell them at the price of *p*_1_. Enterpris’ A's efforts in sales will also form part of the cost. Therefore, enterprise A reasonably increases the market price *p*_2_(1+*x*_*r*_) at the cost of $c_{2}^{0}+z\beta_{f}x_{f}^{2}/2+\beta_{r}x_{r}^{2}/2$, and the consumer demand is still *x*. Enterprise A sells the amount $q-\int_{0}^{q}(q-x)f(x)dx$ of blueberries to consumers within the period of less than the life cycle *T* of blueberries, and after making profits, it allocates profits with farmers at the proportion of (1−*φ*).

Under non-symbiotic conditions, Changbai Mountain blueberry management farmer's planting cost $c_{1}^{s}$ and enterprise A’s processing cost $c_{2}^{s}$ can be obtained from the basic data and formula (7) in the previous article, as shown in Table 1:

**Table 1:** Related cost data of experimental case.

symbol	parameter meaning	data	unit
$c_{1}^{s}$	production cost of planting farmers under symbiotic conditions	9.58	yuan/0.5kg
$c_{2}^{s}$	processing costs of operating enterprises under symbiotic conditions	6.78	yuan/0.5kg
$c_{1}^{o}$	production cost of farmers under non-symbiotic conditions	8	yuan/0.5kg
$c_{2}^{o}$	processing costs of operating enterprises under non-symbiotic conditions	4	yuan/0.5kg
*x*_*f*_	input in production efforts of planting farmers	3	yuan/0.5kg
*x*_*r*_	marketing effort input of the management enterprise	2	yuan/0.5kg
*β*_*f*_	production effort cost parameters of planting farmers	0.7	
*β*_*r*_	the selling effort cost parameters of the management enterprise	0.6	
*z*	the apportionment ratio of production effort cost between the management enterprises and farmers	0.5	

The expected revenue $\pi_{1}^{j},j=o,s$ of blueberry farmers and the expected revenue $\pi_{2}^{j},j=o,s$ of enterprise A at Changbai Mountain before and after the establishment of the symbiotic relationship can be obtained according to the basic data and relevant models in Table 2.

**Table 2:** Related benefit data for experimental case.

symbol	parameter meaning	data	unit
$\pi_{1}^{o}$	the expected income of planting farmers under non-symbiotic conditions	(16)	yuan
$\pi_{2}^{o}$	the expected income of management enterprise under non-symbiotic conditions	(17)	yuan
$\pi_{1}^{s}$	the expected income of planting farmers under symbiotic conditions	(20)	yuan
$\pi_{2}^{s}$	the expected income of management enterprise under symbiotic conditions	(21)	yuan
*T*	active life cycle	30	day
*Q*	the amount of agricultural products acquired by the enterprises from farmers	100	kg
*q*	The actual transportation quality of the products purchased by the management enterprise	(18)	kg
*λ*(*t*)	loss ratio	(1)	
*t*	travelling time	Var.	day
*p*_1_	the price of agricultural products acquired by the enterprise from the farmers	17	yuan/kg
*p*_2_	the actual selling price of the management enterprise	36	yuan/kg
*φ*	profit sharing rate between operating enterprises and farmers	0.8	

Solutions:

$$\left\{ \begin{array}{c} \pi_{1}^{o}=900\\ \pi_{1}^{s}=2970-21.6Q^{*}(t)\\ \pi_{2}^{o}=2500+36Q^{*}(t)-400e^{0.023t}\\ \pi_{2}^{s}=8470+86.4Q^{*}(t)-520e^{0.023t} \end{array}\right.$$ (30)

From Eq (2.16) and (4.1), we can obtain:

$$\left\{ \begin{array}{c} \Delta \pi_{1}^{j}=2070-21.6Q^{*}(t),j=o,s\\ \Delta \pi_{2}^{j}=5970+50.4Q^{*}(t)-120e^{0.023t},j=o,s\\ S_{i}(\left\{N_{1},N_{2}\right\})=8040+28.8Q^{*}(t)-120e^{0.023t} \end{array}\right.$$ (31)

It is easy to know from 0≤*t*≤*T* that the new symbiotic energy *S*_*i*_({*N*_1_,*N*_2_})>0 of the two symbiotic units of Changbai mountain blueberry enterprise A and planting farmers is in line with the practical significance. Moreover, the expected benefits $\Delta \pi_{1}^{j}$ and $\Delta \pi_{2}^{j}(j=o,s)$ before and after the symbiosis relationship are both greater than zero, that is to say, by establishing a symbiotic system, both symbiotic units gain benefits in the interaction relationship, which shows that the symbiotic energy equation established in this paper is effective.

#### Analysis of the singularity distribution of the symbiotic system

**Fig 6:**
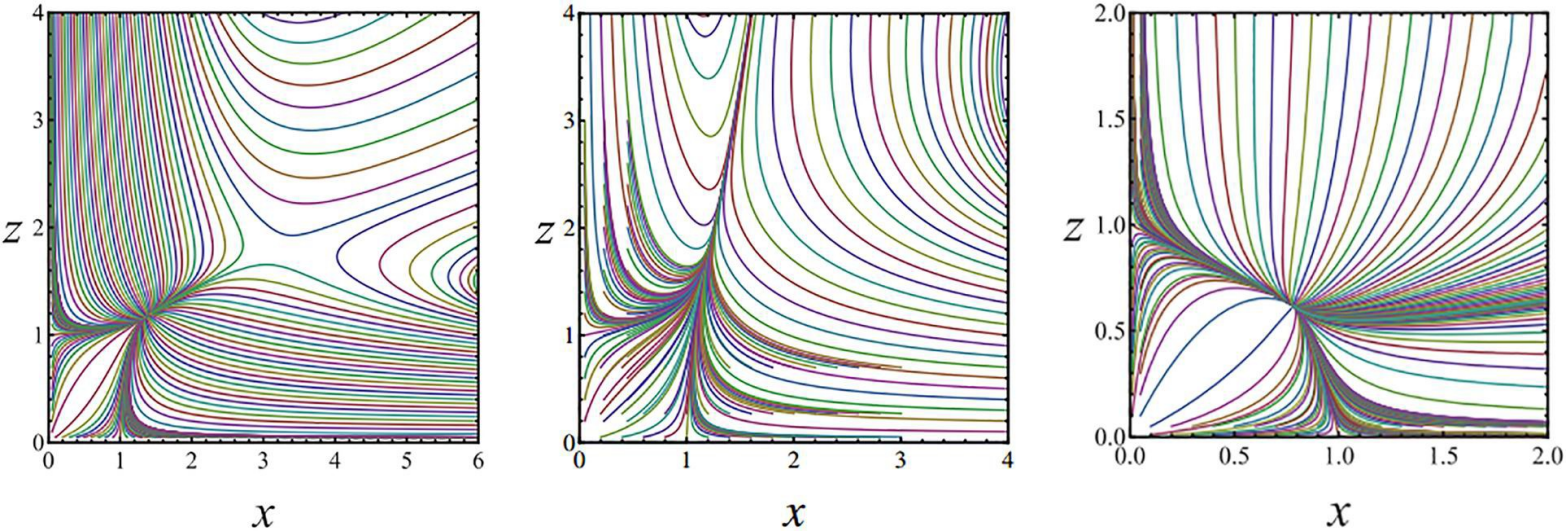
Phase diagrams of symbiotic systems with different symbiotic parameters. (a) Stable degradation node and saddle point phase diagram. (b) Stable degradation node phase diagram. (c) Stable nodal phase diagram.

In the phase diagram of the symbiosis system between enterprise A and farmers, the distribution of the MATLAB numerical simulation track line reflects the distribution of characteristic roots of ordinary differential equation of the dynamic system of this symbiosis system. Under different symbiotic parameters, the phase diagram of the symbiotic system is shown in Fig 6:

①when $b+g=\frac{1}{e},{\left\{b=\frac{1}{2e},g=\frac{1}{2e}\right\}}_{1}$;

②when $b+g=0,{\left\{b=0.2,g=-0.2\right\}}_{2}$;

③when $b+g<0,{\left\{b=-1,g=-2\right\}}_{3}$.

In Fig 6A, the symbiotic parameter satisfies ${\left\{b=\frac{1}{2e},g=\frac{1}{2e}\right\}}_{1}$ when $b+g=\frac{1}{e}$; at this time, the trajectory of *xz* in the phase diagram of the symbiotic system shows two stable points: the first one is equal to the real root stable degenerate node, and the phase diagram is solved. The stable point is {x* = 1.39,z* = 1.178}_1_; the other is the side where the saddle point has a stable point, and the stable point of the phase diagram is {x* = 3.28,z* = 1.811}_2_. Substituting the conclusions into Eq (29), when *b*+*g* = 0, the eigenvalues are *λ*_1_ = −1,*λ*_2_ = −1, which is consistent with the definition of stable degenerate nodes; when {*b* = 0.25,*g* = 0.1}, the eigenvalues are *λ*_1_ = −1,*λ*_2_ = 1.079, which is consistent with the saddle point definition.

In Fig 6B, when the symbiotic parameters satisfy *b*+*g* = 0,{*b* = 0.2,*g* = −0.2}_2_, the trajectories of *x*,*z* in the symbiotic system phase diagram are all tangent to a straight line, and the stability point of the phase diagram is {x* = 1.39,z* = 1.178}, which is the stable degradation node in Fig 6B. This phenomenon indicates that there is no other stable point in the region of $b+g>\frac{1}{e}$, that is, the state is the side with no stable point in the symbiotic saddle point.

In Fig 6C, when the parameter satisfies *b*+*g*<0,{*b* = −1,*g* = −2}_3_, then the trajectory of *x*,*z* in the phase diagram of the symbiotic system presents a stable point, and the stable point of the phase diagram of the symbiotic system is {x* = 0,785,z* = 0.6164}. By substituting the obtained conclusion into Eq (29), the characteristic roots of Lyapunov of stable nodes are solved as *λ*_1_ = −1,*λ*_2_ = −2.4516, which conforms to the definition of stable nodes.

#### Analysis of the evolution direction of the symbiotic system under different symbiotic modes

The basic data of the symbiosis system are shown in Table 3. By Eqs (9), (13) and (14), the total symbiosis energy *E*_*1*_ of blueberry farmers and the total symbiosis energy *E*_*2*_ of enterprise A can be obtained.

**Table 3:** Basic data of experimental case.

symbol	parameter meaning	data	unit
*E*_1_	symbiotic energy of farmers	(9)	yuan
*E*_2_	symbiotic energy of management enterprise	(9)	yuan
*μA*_1_	the carrying capacity of farmers under non-symbiotic conditions	10000	yuan
*μA*_2_	the carrying capacity of management enterprise under non-symbiotic conditions	20000	yuan

Solutions:

$$\left\{ \begin{array}{l} E_{1}=100000+b(8040+28.8Q^{*}(t)-120e^{0.023t})\\ E_{2}=200000+g(8040+28.8Q^{*}(t)-120e^{0.023t}) \end{array}\right.$$ (32)

When $b+g>\frac{1}{e}$, there is no singularity in the symbiosis system, and under the mutual-symbiosis mode, the economic output indicators*N*_1_ and *N*_2_ of enterprise A and planting farmers are in the infinite exponential growth mode, and the symbiosis energy and expected benefits formed by the two symbiosis units in the system are all greater than the non-symbiosis state, which is the ideal state in reality. When $0\leq b+g\leq \frac{1}{e}$ or *b*+*g*<0, The initial value is {*N*_1_,*N*_2_}_0_ = {0.01,5}. When different symbiotic parameters converge to a stable state, the data are shown in Table 4, and the analysis conclusion is as follows:

**Table 4:** The economic indicators data table of Changbai Mountain blueberry management enterprise A and planting farmers.

the symbiotic parameter important indicators	$0\leq b+g\leq \frac{1}{e}$					*b*+*g*<0
	*b* = 0.25,*g* = 0.1			*b* = −0.75,*g* = 1		*b* = −1,*g* = −2
	*t* = 20	*t* = 15	*t* = 10	*t* = 15	*t* = 10	*t* = 10
$\Delta \pi_{1}^{j},j=o,s$	1171.59	799.89	468.89	799.89	468.89	468.89
$\Delta \pi_{2}^{j},j=o,s$	8011.09	8892.18	9679.06	8892.18	9679.06	9679.06
*E*_1_	11709.9	12023.1	12302.6	3930.79	3092.14	789.52
*E*_2_	20683.9	20809.2	20921.1	11907.7	10789.5	1579.04
{*N*_1_,*N*_2_}	{0.342,4.177}	\	\	{1.668,1.227}	\	{0.788,0.303}
Δ*N*_1_	0.341	\	\	1.667	\	0.787
Δ*N*_2_	-0.823	\	\	-3.773	\	-4.697

1. Expected return $\Delta \pi_{1}^{j}$ and $\Delta \pi_{2}^{j}(j=o,s)$
  - Both $\Delta \pi_{1}^{j}$ and $\Delta \pi_{2}^{j}(j=o,s)$ are greater than 0, and the economic profit of both enterprise A and farmers has been improved; Stable sales channels drive farmers' economic returns, and farmers' expected returns $\Delta \pi_{1}^{j}$ increase with time *t*;
  - To improve the quality of fresh agricultural products and qualified production, enterprise A needs to pay part of the income, production and sales input cost, then the expected income $\Delta \pi_{2}^{j}(j=o,s)$ of enterprise A decreases with the growth of time *t*. This helps maintain stable, long-term symbiosis.
2. Symbiosis energy *E*_1_ and *E*_2_
  - In the mutual-symbiosis model (*b*>0,*g*>0), the symbiosis energy *E*_1_ and *E*_2_ produced by enterprise A and farmers are higher than the two parasitic models, which proves the conclusion that mutual-symbiosis is the optimal development state of the symbiosis system. *E*_1_ and *E*_2_ decrease with the increase of time, indicating that the shorter the time, the higher the market value of fresh agricultural products, reflecting the direct impact of the characteristics of fresh agricultural products on the income of farmers and enterprises.
  - Under the parasitic mode (*b*<0,*g*>0), enterprise A and farmers need to maintain a stable and sustainable cooperation state to obtain more symbiotic energy, which promotes their development into a mutualistic symbiotic mode with a slower convergence rate. This phenomenon confirms the conclusion that mutualism is the ultimate direction of symbiotic system development.
3. Index of economic output of symbiotic system Δ*N*_1_ and Δ*N*_2_
  - In the mutualistic symbiosis model (*b*>0,*g*>0), when observing *t* = 20,*t* = 15, and *t* = 10, the symbiosis system is convergent to a steady state, {*N*_1_,*N*_2_} has no solution in *t* = 15 and *t* = 10, and a long and stable cooperative relationship can help the mutualistic symbiosis model play a role.
  - Under different symbiosis modes, the convergence speed of the symbiosis system between enterprise A and farmers is different. The mutualism symbiosis model converges slowly, the economic output index is good; The parasitic model converges quickly, and the index of economic output is poor. The initial economic output index is set as {*N*_1_,*N*_2_}_0_ = {0.01,5}. Under the mutualistic symbiosis mode, the symbiosis system converges to stability at *t* = 20, at this time {*N*_1_,*N*_2_}_*f*_ = {0.342,4.177}. In the parasitic mode (*b*<0,*g*>0), the symbiotic system converges to stability at *t* = 15, at this time {*N*_1_,*N*_2_}_*f*_ = {1668,1.227}. In the other parasitic mode (*b*<0,*g*<0), the symbiotic system converges to stability at *t* = 10, at this time {*N*_1_,*N*_2_}_*f*_ = {0.788,0.303}.
  - The change of the economic output index Δ*N*_1_ of farmers increases under the three modes, while the change of economic output index Δ*N*_2_ of enterprise A decreases. To obtain long-term high-quality and reliable supply channels, enterprises need to give assistance to farmers, resulting in the decline of their economic output index. However, the long-term symbiotic relationship brings more symbiotic energy to the enterprise, which can gain more profits in the fresh agricultural products market and improve the overall competitiveness of the symbiotic system.

## Conclusions

In this paper, symbiotic theory is applied to study the symbiotic system and stability between fresh agricultural products management enterprises and farmers. The research work, research conclusions and results are summarized as follows:

1. Based on the symbiotic system principle, the symbiotic unit model, symbiotic model, symbiotic energy model and symbiotic environment model are respectively established in this paper.
2. In this paper, Lyapunov method is used to analyse the stability of symbiotic system.
3. The improved symbiosis system stability analysis method proposed in this paper, which is oriented towards criterion quantification, solves the problem that the symbiosis relationship and evolutionary law of the fresh agricultural products supply chain lack, to some extent, the support of a quantification method. Combined with numerical examples, MATLAB numerical simulation results show that the symbiotic energy model is effective and helpful for depicting and revealing the dynamic mechanisms of the symbiotic system.

Lyapunov indirect method was used to determine the type of symbiosis pattern by analysing the positive and negative symbiosis parameters. The simulation results are consistent with the theoretical results. Compared with the parasitic mode, the mutualistic symbiosis mode can generate more symbiotic energy and prolong the convergence time of the symbiosis system, so that the economic output index of the symbiosis system under a stable state can reach the optimal level.

The above research results provide a quantitative theoretical basis for the choice of cooperative relationship between enterprises and farmers, promote the formation of a true sense of symbiosis, and provide a path reference for the whole fresh agricultural product supply chain to improve the value-added space. Compared with the qualitative analysis based on the economic form, this paper provides a scientific quantitative standard for the stability of the symbiotic relationship in the supply chain of fresh agricultural products.

The influencing factors of the symbiotic energy model are limited to the symbiotic parameters *b*,*g*. Future follow-up studies will consider the influence of other parameters of the symbiotic energy model and parameter combination optimization on the stability of the symbiotic system, to more comprehensively describe the evolutionary process of the symbiotic system and reveal the complex relationships between symbiotic units.
